# Molecular-targeted therapy for advanced anaplastic thyroid cancer combined with nutritional support

**DOI:** 10.20407/fmj.2018-003

**Published:** 2018-12-06

**Authors:** Yuka Maegawa, Takashi Higashiguchi, Akihiko Futamura, Norimasa Tsuzuki, Miyo Murai

**Affiliations:** 1 Department of Pharmacy, Fujita Health University Nanakuri Memorial Hospital, Tsu, Mie, Japan; 2 Department of Surgery and Palliative Medicine, Fujita Health University, School of Medicine, Toyoake, Aichi, Japan

**Keywords:** Thyroid carcinoma, Anaplastic thyroid cancer, Molecular-targeted therapy, Lenvatinib, Nutritional support

## Abstract

Management of anaplastic thyroid cancer (ATC) is often difficult because of its aggressive characteristics. Molecular-targeted therapy was recently introduced as an alternative therapeutic strategy for ATC; lenvatinib is a molecular-targeted agent that is currently indicated only in Japan for the treatment of ATC. Here we report the case of an 86-year-old Japanese woman with ATC who was treated with lenvatinib at our hospital and exhibited a remarkable response. Computed tomography showed tumor shrinkage by day 8 and stable disease until day 32. She maintained activities of daily living (ADLs) until shortly before her death. The patient’s resting energy expenditure and body composition were analyzed at the time of admission. Potential toxicity risk of lenvatinib was evaluated based on these data. Enteral nutrition for oral intake was supplied to compensate for her lack of dietary intake and to improve metabolism for the purpose of suppressing lenvatinib toxicity. She also engaged in physical rehabilitation to avoid developing sarcopenia, which is thought to be a risk factor of molecular-targeted therapy toxicity, and to maintain her activity level. We emphasize the importance of a team approach for providing an appropriate treatment regimen to maintain ADLs, which includes nutritional support, physical rehabilitation, and aggressive therapy with lenvatinib.

## Introduction

Anaplastic thyroid cancer (ATC) is a rare and extremely aggressive malignancy, with a median survival of ≤6 months. A standard treatment strategy for ATC has not been established. Molecular-targeted therapy was recently introduced as an alternative therapeutic strategy for ATC. In March 2015, lenvatinib, a newly developed tyrosine kinase inhibitor, was approved for the treatment of ATC in Japan. This treatment is currently only approved in Japan. Although lenvatinib treatment requires close monitoring for development of adverse events such as hypertension, fatigue, proteinuria, and nausea, it has shown significant clinical benefits for ATC patients.

Our team treated a terminal-stage ATC patient with lenvatinib combined with nutritional support, physical rehabilitation and careful monitoring for adverse events. She exhibited a good response to lenvatinib treatment and maintained her activities of daily living (ADLs) until shortly before her death. The patient’s family provided written informed consent for her case details to be published.

## Case report

An 86-year-old Japanese female presented at our hospital with ATC (cT4bNxM1, stage IVc).^1^ Surgical resection was considered impossible because of multiple lung metastases. Performing a tracheotomy was also considered an ineffective option because of subglottic narrowing. The patient was thus referred to our hospital for palliative care 19 days after diagnosis.

The patient and her family expressed a strong desire for the patient to engage in active therapy and extend her life. The patient planned to go on trips with her family about once a month, and she hoped to enjoy these trips in good physical condition. Thus, she underwent molecular-targeted therapy aimed towards reaching her goal.

**Figure 1:**
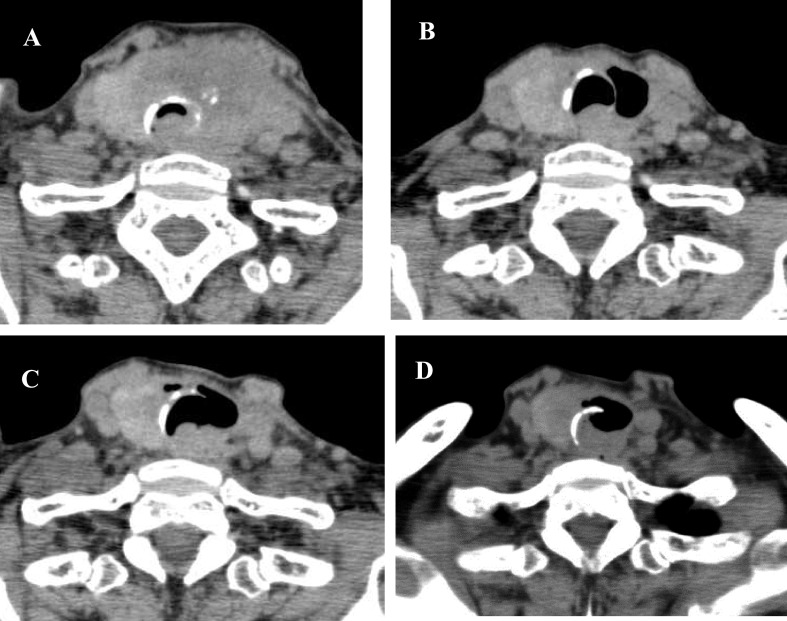
CT scans of patient’s neck. **A:** First day of hospitalization. **B:** Day 8, tumor shrinkage observed. **C:** Day 20, primary tumor stability observed. **D:** Day 47, growth of the original lesion observed.

At the time of admission, the patient had dyspnea, dysphagia, hoarseness, and sore throat. Her treatment was initiated with 24 mg/day of lenvatinib mesilate capsules on the first day of hospitalization. Morphine hydrochloride hydrate and loxoprofen sodium hydrate were administered to relieve symptoms of dyspnea and sore throat. In the cervical computed tomography (CT) examination on day 8, a significant reduction was seen in the volume of the original lesion (Figure 1). The patient’s hoarseness had disappeared by day 14, and treatment with morphine hydrochloride hydrate was discontinued on day 15 because dyspnea was no longer present. An elevation of blood pressure was observed as a side effect of lenvatinib but was controlled by antihypertensive medications.

On day 20, blood and urine tests detected a decreased platelet count (grade 1: Common Terminology Criteria for Adverse Events (CTCAE) ver. 4.0), increased aspartate aminotransferase (grade 1: CTCAE ver. 4.0), hematuria (grade 1: CTCAE ver. 4.0), and elevated TSH. Levothyroxine sodium hydrate was added to the patient’s treatment on day 21, and the dosage of the lenvatinib mesilate was reduced to 20 mg/day to manage these side effects. Cervical CT at that time showed stability of the original lesion (Figure 1).

On day 25, palmar-plantar erythrodysesthesia syndrome (PPE) (grade 1: CTCAE ver. 4.0) was observed. The urea ointment used for the prevention of PPE, initiated at the time of admission, was continued, and difluprednate ointment was added to reduce the symptoms of PPE, which then improved. The patient’s sore throat gradually subsided and on day 29 loxoprofen was discontinued.

**Figure 2:**
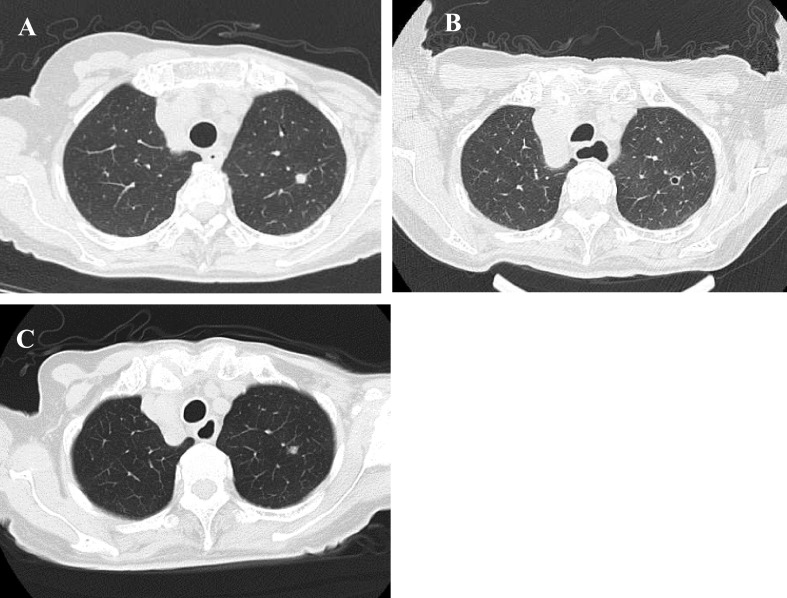
CT scans of patient’s chest. **A:** First day of hospitalization. **B:** Day 32, regression of pulmonary lesions observed. **C:** Day 47, recurrence of pulmonary lesions observed.

A chest CT performed on day 32 revealed regression of the pulmonary lesions (Figure 2).

The patient’s resting energy expenditure (REE) was measured using a Vmax SPECTRA 29n^®^ (SensorMedics Corp., California, USA) indirect calorimetry at the time of admission and on day 29. At the time of admission, the measured REE was 971 kcal/day (Harris-Benedict equation REE prediction: 993 kcal/day). The patient’s dietary intake was approximately 500 kcal at the time of admission. Ensure Liquid-H^®^ (a liquid enteral product containing 375 kcal/pack) was administered at one pack per day to compensate for the patient’s lack of dietary energy intake. Inner Power^®^, a 139 kcal/pack functional dietary supplement (FDS) consisting of Coenzyme Q10, branched-chain amino acids, citric acid, L-carnitine, zinc and, various vitamins, was also administered at one pack per day to suppress progression of cancer cachexia.

**Figure 3:**
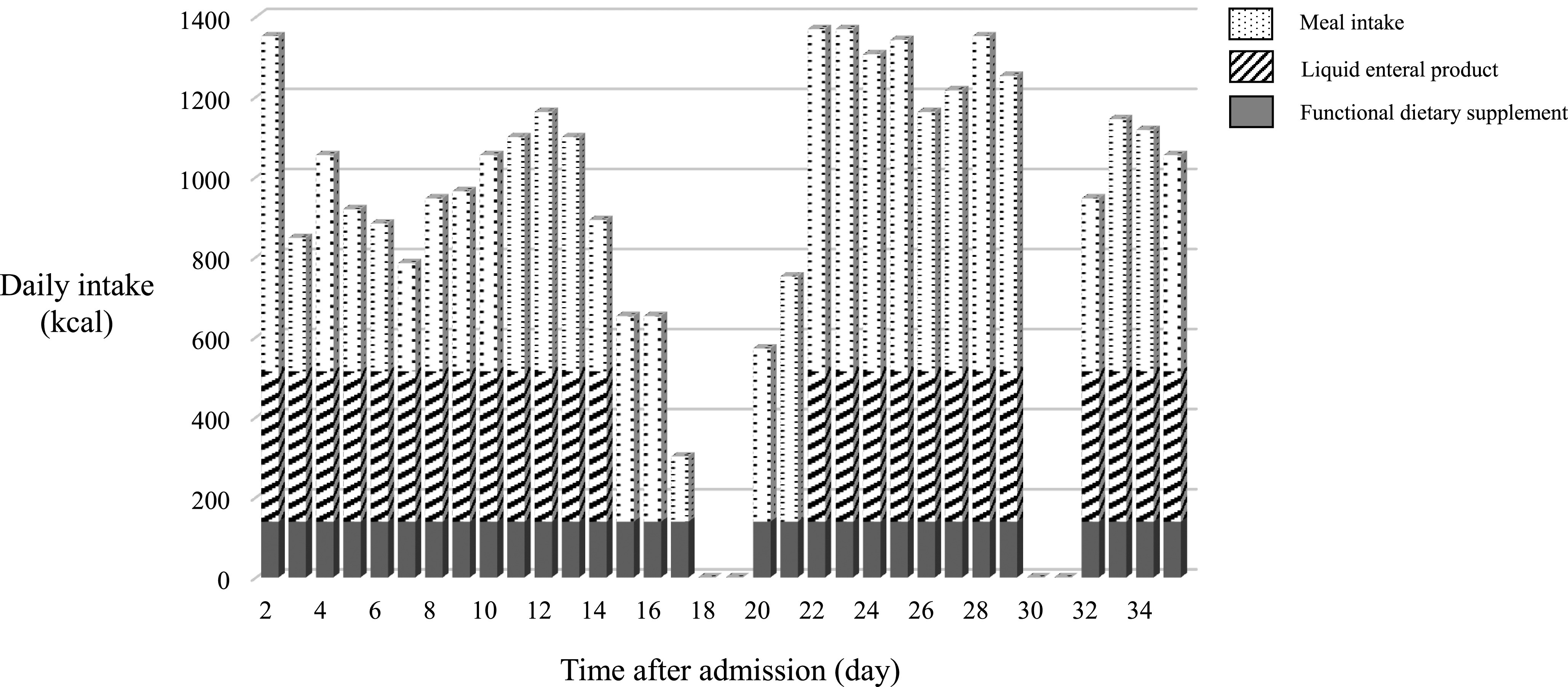
Patient’s meal intake course. Of note, the patient stayed outside the hospital from day 18 to 19, and from day 30 to 31 to attend trips with her family.

The patient also engaged in physical rehabilitation toward the goal of maintaining her activity level (Table 1). Stable meal intake of 1200 kcal became possible after day 22 because pain on swallowing subsided as a result of lenvatinib treatment (Figure 3). On day 29, the measured REE was 922 kcal/day (Harris-Benedict equation predicted REE: 984 kcal/day). The respiratory quotient (RQ) calculated using indirect calorimetry on the first day of hospitalization and on day 29 were 0.79 and 0.95, respectively. The patient’s transthyretin was increased from 9.9 mg/dL at the time of admission to 18.4 mg/dL on day 29.

The patient’s body composition was analyzed using an Inbody S10^®^ (Inbody Japan Inc., Tokyo, Japan) body composition analyzer. Her soft lean mass was 27.9 kg on the first day of hospitalization and 28.4 kg on day 29 (Table 2). She was able to maintain her ADL level as shown by Functional Independence Measure with 123 total points both at the time of admission and at discharge on day 36.

After discharge, the patient was able to spend quality time at home with her family and went on a trip with them in good physical condition. However, cervical and chest CTs on day 47 revealed growth of the tumor (Figure 1, Figure 2). The lenvatinib dose was increased to 24 mg/day to control tumor growth, and oxycodone tablets were prescribed to reduce throat pain. Tumor growth could not be controlled, however, and she was rehospitalized on day 53. She developed respiratory failure after rehospitalization and became unable to take lenvatinib on day 54. Her respiratory condition was further aggravated by the respiratory tract obstruction. She developed somnolence on day 56 and passed away on day 60.

## Discussion

The orphan disease, ATC, is a highly lethal form of thyroid cancer. Disease-specific overall survival rate of ATC patients at 6 months has been reported as 36%, 31.6%, and 37%.^2–4^ Despite great efforts to improve ATC treatment, the prognosis of ATC remains poor. Molecular-targeted therapy was introduced as an alternative therapeutic strategy for highly malignant cancers. Although progression toward development of targeted therapies for ATC is slow, these therapies represent a new approach to the treatment of ATC.^5,6^ Takahashi et al. reported a phase II trial using lenvatinib.^7^ In that study, the ATC cohort consisting of 11 patients showed three partial responses, seven cases of stable disease, and one case of progressive disease. Lenvatinib is currently approved in Japan for all types of thyroid cancer including anaplastic, whereas it is approved only for the treatment of differentiated thyroid cancer in other countries. The option of lenvatinib treatment was suggested to our patient based on these data.

Pharmacists should carefully evaluate clinical evidence and patient needs when deciding whether a medication is appropriate, this was followed when deciding on a course of therapy for our patient. The patient had a strong desire to extend her life and improve her physical condition, therefore we felt treatment with lenvatinib was appropriate.

We are sometimes conflicted regarding palliative care. Some patients are told by their physicians to forego further treatment for tumor reduction and undergo only palliative care, based on the condition of the patient’s body. However, some of these patients may be candidates for further effective chemotherapy. These patients tend to think of palliative care as a form of “acceptance” and choose to forego further chemotherapy. Mori et al. published a report discussing possible approaches to cancer chemotherapy in palliative care. The report noted the importance of emphasizing the wishes of patients and their families, and working together to achieve them.^8^ We propose that medical staff should endeavor to understand each patient’s true feelings, which may be hidden under the guise of “acceptance.”

In our patient’s case, all of the attending medical staff shared the patient’s goal of her living as long as possible, and enjoying a trip with her family in good physical condition. The treatment goals were thus set not only to extend the patient’s life but also to improve her quality of life. Our team expected a good response to lenvatinib treatment but did not ignore the possibility of adverse effects; special attention was paid to avoid such developments. The dose of lenvatinib was decreased before any grade 2 adverse events developed following the treatment strategy of a doctor on our team who has shown that, in most cases, lenvatinib is decreased after grade 2 adverse events. We felt it was appropriate to continue administration of lenvatinib as long as possible to meet the patient’s main desire to extend her life. In a previous study, lenvatinib demonstrated manageable toxicities with dose adjustments in patients with ATC.^9^ In the present case, toxicities were manageable with dose adjustment until termination of lenvatinib administration. This case indicated that prompt dose adjustment based on careful monitoring of symptoms is necessary to maintain the patient’s quality of life. Pharmacists contributed to early detection of side effects by providing pertinent information to medical personnel regarding frequent side effects which should be monitored, including optimal time for blood and urine sampling. We also contributed to prevent worsening of lenvatinib side effects by suggesting the application of both urea ointment and difluprednate ointment to improve PPE symptoms, as well as use of antihypertension medications for controlling blood pressure.

Another important role for pharmacists is to evaluate the potential toxicity risk of anticancer agents and provide suggestions for suppressing toxicity. Jouinot et al. investigated whether REE measurement before initiating anticancer treatments could predict the occurrence of early limiting toxicity; they reported that toxicity was associated with abnormal metabolism.^10^ It was also reported that sarcopenia was a risk factor for molecular-targeted therapy since it alters pharmacokinetics and increases exposure to anticancer drugs.^11,12^ We predicted our patient’s case to have less risk of toxicity because of the normal basal energy metabolism shown by the ratio of measured REE using indirect calorimetry and the REE calculated by the Harris-Benedict equation. However, since our patient had a low level of soft lean mass, increasing her skeletal muscle mass seemed necessary for reduction of toxicity. Thus, we suggested the use of Ensure Liquid-H^®^, the most highly concentrated enteral nutritional product approved as a medicine, for maintaining her nutritional state and preventing muscle reduction. It was chosen for ease of consumption and the small volume needed for treatment.

Inner Power^®^, an FDS, was also administered to suppress the progression of cancer cachexia. We previously investigated the clinical effects of FDS on terminal cancer patients; our findings demonstrated that the use of an FDS reduced the occurrence of clinical symptoms and promoted the recovery of physical function, while moderating the morbid metabolism associated with cancer.^13^ We also observed the effects of an FDS in tumor-bearing mice and reported the possibility that oral administration of an FDS suppresses tumor growth and metastasis.^14^ Thus, FDS was chosen as part of her treatment plan. Physical rehabilitation was prescribed as well to avoid development of sarcopenia.

Oral consumption of a liquid enteral product and FDS, along with physical rehabilitation, allowed our patient to maintain her muscle mass and avoid developing sarcopenia. In addition, adequate intake of nutrients improved the patient’s metabolism as shown by the RQ data. In her case, toxicity could be controlled by treatment efforts including nutritional support and physical rehabilitation, resulting in maintenance of the patient’s level of ADLs and her quality of life. Thus, nutritional support may play a major role in the treatment of cancer patients, as observed in the present case.

Because lenvatinib was only recently approved in Japan, there are few reports concerning the use of lenvatinib for ATC.^15,16^ To our knowledge, there are no reports discussing the course of ADLs for ATC patients. The present case shows the efficacy of the combination of lenvatinib treatment with nutritional support and physical rehabilitation in regard to maintaining ADLs.

In conclusion, this case report demonstrates the effectiveness of lenvatinib treatment combined with nutritional support and physical rehabilitation, performed by a medical team, in maintaining ADLs of a terminal-stage ATC patient. We suggest ‘hybrid palliative care’ as a new approach to caring for terminal cancer patients, including those with ATC. ‘Hybrid palliative care’ should be provided by a medical team and consist of a combination of aggressive antitumor therapy plus all appropriate types of supportive care, including nutritional support, to improve quality of life and enable the best life for the patient until death.

## Figures and Tables

**Table1:** Rehabilitation activities

Time after admission (day)	Rehabilitation Activities^a)^
8	Evaluation of activity level: 40 min

11	Standing-up motion: 15 min Walking: 15 min Standing postural balance: 10 min

13	Standing-up motion: 10 min Walking: 20 min Stair stepping: 10 min

14	Standing-up motion: 10 min Walking: 20 min Stair stepping: 10 min

19	Muscle strengthening exercise: 20 min Walking: 10 min Stair stepping: 10 min

29	Muscle strengthening exercise: 20 min Walking: 20 min

^a)^ In addition to prescribed rehabilitation activities, the patient underwent self-rehabilitation daily.

**Table2:** Body composition measurements

		Day of admission	Day 29
Weight (kg)		49.5	48.0
Soft Lean Mass (kg)		27.9	28.4
Body Fat Mass (kg)		19.7	17.6
Segmental Lean Analysis (kg)	Right Arm	1.30	1.26
	Left Arm	1.25	1.26
	Trunk	13.6	13.4
	Right Leg	4.11	4.23
	Left Leg	4.23	4.39
